# Useful and Useless Reading

**Published:** 1873-04

**Authors:** 


### USEFUL AND USELESS READING.

An immense amount of reading, worse than worthless, is daily scattered over the country in books, pamphlets, newspapers and magazines, in the shape of trashy stories of the blood and thunder kind, with "illustrations" to match, making the minds of children early familiar with sights and scenes horrible, indecent and profane. Very many religious newspapers devote whole columns to impossible or inane stories, instead of making scripture narrations "classic," as many of the reputed facts of history were thus made classic by Homer and Horace and Virgil and others. Many a young man leaves college, and many a young lady leaves her boarding school, more familiar with the myths and legends of profane history than the truthful narrations of the Bible. Let cultivated and thoughtful parents make a change at once; have less of the novel and more of fact; let each child be a subscriber to some really useful periodical, practical and scientific, affording instruction as to the common affairs of life. It is impossible for any intelligent parent to take up a single number of such papers as the *Scientific American* or *Boston Journal of Chemistry*, edited with so much judgment and ability by Dr. Nichols, without finding a great variety of articles abounding in useful information, interesting and instructive; the knowledge of which elevates and refines, and dignifies.

